# Epidemiological aspects of new HIV/AIDS diagnoses in south-east Romania

**DOI:** 10.1186/1471-2334-14-S2-P53

**Published:** 2014-05-23

**Authors:** M Arbune, C Georgescu, D Tutunaru, M Dobre, A Nechita

**Affiliations:** 1 "Dunarea de Jos" University, Galati, Romania

## Introduction

The characteristic of HIV/AIDS Romanian epidemic is the high proportion of youth infected in 1988-1990, during their first year of life, when a nosocomial epidemiological accident took place. In Galati (South-East Romania) they were recorded 535 people with HIV/AIDS during 1990-2003 and 79.5% belonged to the pediatric nosocomial cohort. The mortality was 56.8% and half of the deaths were recorded during the first year after diagnosis. The aim of the study is to identify the changes in the HIV/AIDS epidemic in the last 10 years.

## Materials and methods

This retrospective study was based on epidemiological reports of the newly diagnosed HIV/AIDS patients. The descriptive statistics and comparative Kaplan-Meier graphs by transmission way were analyzed using XL-Stat Software.

## Results

During 2004-2013, 182 new patients have been diagnosed with HIV/AIDS in Galati. Most patients are young people with the same age as the pediatric cohort of HIV/ AIDS, but predominance of heterosexual transmission marks the change of the epidemic pattern. (table 1). Late presentation is less associated with sexual than other transmission ways (p=0.026), but is most likely expected in males (p=0,003), with symptomatic context (p<0,001). Death rate is 15%. Retention in care was achieved in 95% of cases. Tuberculosis is still the leading indicator of AIDS (21.4%). Tuberculosis (12/26) and cancer disease (6/26) are the main causes of death.

**Table 1:** Characteristics of new HIV/AIDS diagnosed patients in Galati

Median diagnostic age:	23[1;78]
Sex: Male/ Female	98/84

Area: Urban/ Rural	87/95

Transmission:	

Perinatally	4.4%

Nozocomial	19.8%

Heterosexual	56%

MSM	1.6%

IVDU	3.8%

Unknown	14.3%

Context of HIV testing:	

HIV partner	17.6%

Pregnancy	11.5%

Perinatal exposure	3.6%

Drugs users	1.6%

Other screening programs	8.2%

*Symptomatic:*	*51.2%*

Clinical CDC-class: A/B/C	19/81/83

Immunological-class:1/2/3	27/52/103

## Conclusions

The life expectancy of HIV/AIDS patients has obviously improved in the last decade, but the yearly number of new cases is constant, with a high rate of late presenters. The strategy of future screening programs should consider the trends of HIV/AIDS epidemic in our region.

